# Yoga practices in Social Anxiety Disorder (SAnD): A case report WSR to paruresis

**DOI:** 10.1016/j.jaim.2022.100622

**Published:** 2022-09-07

**Authors:** Danish Javed, Shweta Mishra

**Affiliations:** Department of AYUSH, All India Institute of Medical Sciences, Bhopal, Madhya Pradesh, 462020, India

**Keywords:** Case report, Yoga practices, Social anxiety disorder

## Abstract

Social anxiety disorder has characteristic features of the overwhelming anxiety and apprehensions especially in public gatherings. There is always a false perception in mind that someone is continuously watching, noticing or judging their activities. Many of times the situation is associated with some unusual behavioral problem like shy bladder syndrome, in which the person even cannot urinate in public restrooms. In the present case report, a patient was suffering from paruresis-specific social phobia. After administration of *Yogasana, Pranayam, Omkar chanting* and *meditation* practices by him under the supervision of yoga experts, significant improvement was noticed in multiple parameters. Subjective assessment on different scales was done before and after *yogabhyasa*. By regular yoga practices Liebowitz Social Anxiety Scale (LSAS) score was reduced 60.97%, Social Phobia Inventory (SPIN) score was reduced 56.25%, Shy Bladder Scale (SBS) score was reduced up to 59.42% and WHO Quality of life questionnaire- WHO QoL- BREF score was improved 56.81% in physical domain (D1), 34% in psychological domain (D2), 10.34% in social relationship domain and 27.91% in environment domain (D4). Hence, it can be concluded that yoga and meditation practices may play a good role in Social anxiety cases.

## Highlights

• Paruresis-specific social phobia is rare clinical entity. Here, we managed such a case fighting with this specific problem.
• We administered a specific tailored yoga and meditation module for six months.
• We found improvement in Liebowitz Social Anxiety Scale (LSAS) score, Social Phobia Inventory (SPIN) score, Shy Bladder Scale (SBS) score and WHO QoL- BREF score.

## 1 Introduction

Social Anxiety Disorder also called Social Phobia, is portrayed by overwhelming nervousness in regular circumstance which occasionally maintained a strategic distance from because of fright of being watched or investigated by others or acting in a humiliating manner [1]. The patient's anxiety might be centred on side effects like palpitations or feeling faint and is regularly connected with auxiliary apprehensions of biting the dust, losing control or going distraught. Specific phobia situational type, is depicted as a tireless fear that is over the top or preposterous signalled by the nearness or expectation of a particular item or circumstance, for example, open transportation, burrows, spans, lifts, flying, driving or encased spots [2]. Paruresis or shy bladder syndrome a relatively less common condition has been found associated with anxiety and depression. Individual faces trepidation and failure to urinate in open bathrooms when different people are available or may go into the room. Its psychophysiology and management is still less understood and need more to explore [3]. It can be defined as alludes to the failure to start or continue urination in circumstances where there is an impression of genuine or expected examination from others, joined by huge dread and nervousness, shirking of dreaded circumstances, and ensuing negative effect on psychosocial working [4]. It appears as distinct clinical entity or may be intermingled with Social phobia [5]. Many tools like Liebowitz Social Anxiety Scale (LSAS), Social Phobia Inventory (SPIN), shy bladder scale (SBS) have been validated to measure the extent of paruresis [6]. The present case report has been prepared as per the guidelines of CaRe recommendation. An informed written consent was obtained from the patient for reporting this case.

## 2 Case presentation

### 2.1 Patient information

The case study presented here outlines a course of Yoga therapy for a man, who we will call “Mr. A″, presenting with Social Anxiety Disorder with paruresis in a 32 years old, married male, thin built and a confirmed case of “Social Anxiety Disorder”. He was taking regular consultation and treatment from psychiatry OPD of a district hospital. Since, the patient was not getting significant relief in his symptoms, especially in paruresis, he was advised to take consultation from Yoga OPD for his unresolved anxiety and paruresis issues. He visited in Yoga OPD on 1st October, 2018 in AYUSH department at our multispecialty tertiary care center situated in central India region.

### 2.2 Present medical history

The male patient was under psychiatric consultation and treatment for above problems since almost last more than one year i.e. since 03.08.2017. When he came to the yoga unit of AYUSH department, a detailed history was recorded. He was feeling shy and hence his wife helped him in explaining the ailment. He later was comfortable to have a conversation more frankly. He had many complains such as- he was not able to sleep properly in his home in darkness with lights off, difficulty to convey his thoughts and feelings to others, a strong feeling of embarrassment and problem of over thinking. He also had complains of fear and anxiety while being alone at home. He found it difficult to initiate the talk with others, becoming stressful without any specific reason. Specially, he had difficulty in urination at public toilets i.e. paruresis (bladder shyness). He had much difficulty in going to crowded places i.e. agoraphobia. He gradually started suffering from these symptoms since the age of 12 years, but remained unaware of these happenings.

### 2.3 Medical history

The subject had no significant medical or surgical past history. No history of any psychological trauma or any reactive episode was reported by the patient. He has normal physical and mental development since childhood. His parents were very caring and during school time he was a good performer.

### 2.4 Yogic interpretation of the case

Since yoga is not merely the name of physical exercise, it also covers the psychological aspect of well being. In yogic philosophy, all the diseases are originated from the brain itself. It is our “Ego” which plays a very balanced role in development of all types of diseases, whether physical or mental. The cause of disease lies in *avidya*, imbalance in *tridoshas*, ignorance, and excess stress.

विपर्ययो मिथ्याज्ञानमतद्रूप् प्रतिष्ठम् | योगसूत्र १/८.

Mental illnesses are more common in fearful and sad people, improper diet habit, not following balanced life style, low self confidence level, and facing psychological trauma. In such persons, predominantly-*vata* and aggravated *raja* and *tama doshas* easily affect the *manovahi srotasa* and results many types of psychological disturbances.

रजः पित्तनिलाद् भ्रमः। Sushruta sharir 4/56.

Yoga is not only the way of life; but also, can be used for therapeutic, non-pharmacological intervention to treat health issues by experts. *Pranayam*, an integral part of yogic therapeutic modality deals with the rhythmic respiratory cycles. Many studies have shown the beneficial effects of *pranayam* in regulating autonomic nervous system especially by improving heart rate variability [7]. The para-sympathic activation plays key role in controlling anxiety issues. It also establishes a better understanding of person about society and his surrounding environment.

The patient was not responding to standard allopathic pharmacological treatment and psychiatric counseling. He was diagnosed as a case of *Manodaurbalya* and was planned to administer tailored yogic sessions in standard manner. The various subjective parameters on Liebowitz Social Anxiety Scale (LSAS), Social Phobia Inventory (SPIN), Shy Bladder Scale and WHO Quality of life questionnaire- WHO QoL- BREF were recorded systematically during six month of study period. The result was evaluated in terms of percentage change in their values.

### 2.5 Diagnosis

The diagnostic and statistical manual –IV (DSM –IV) states Social phobia is defined as a marked and persistent fear of social or performance situations in which embarrassment may occur. The diagnosis of the patient was made on the basis of symptoms present.

### 2.6 Timeline

**Fig. 1:**
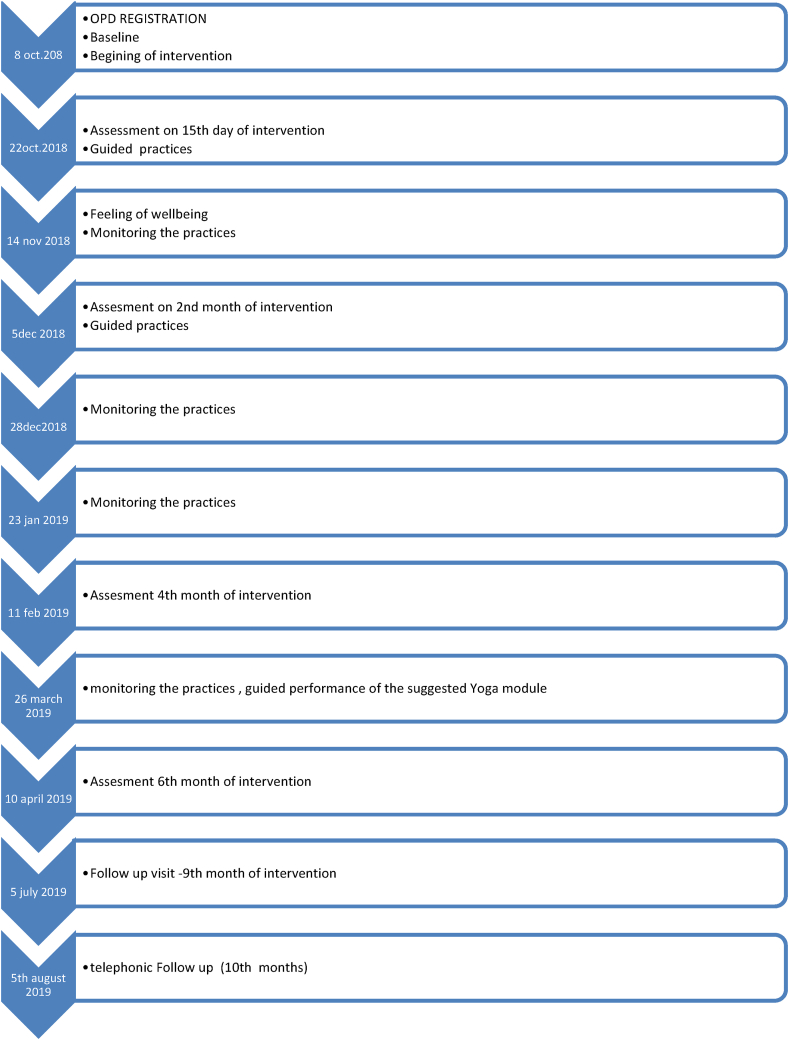
Timeline of case report.

A detail of the study of the patient in hospital from registration to follow up has been given in figure (See:Fig. 1: Timeline of case report).

### 2.7 Intervention

#### 2.7.1 Yoga practices

Patient was convinced to perform the advised guided Yoga practices along with life style modification and stress management. Patient was provided with a “Yoga Practice Module” consisting of *(i) Pranayam, (dirghaswash, bhramari, nadishuddhi), (ii) asanas (Simhasana, veerasana, baddhakonasana, markatasana, viparitkarni), (iii) suryatrataka* (at the time of dawn) and *(iv) OM kara* meditation with *breath awareness*. He was advised to practice the entire intervention mentioned above, for 30 min daily in early morning at his home regularly (See:Table 1 Yoga and meditation protocol).

**Table 1:** Yoga and meditation protocol.

Pranayam	Dirghaswash, Bhramari,Nadishuddhi	15 min approx.
*Asanas*	*Simhasana, Veerasana, Baddhakonasana, Markatasana, Viparitkarni*	10 min *approx.*
Meditation	*OM kara* meditation with *breath awareness*	05 min *approx*
*Surya trataka*	*-*	At the time of dawn

#### 2.7.2 Diet modifications

Diet is an integral part of health. It not only deals with physical health but also mental and spiritual health. *Ahaar parinaam* or digestive metabolites bring a drastic change in *sharirik* (bodily humors) and *mansik guna* (mental humors) both. *Sata, Rajah* and *tamasguna* are responsible for psychological well-being. *Satvik ahaar* can nullify the undue stress, anxiety and depression [11].

**युक्ताहारविहारस्ययुक्तचेष्टस्यकर्मसु| युक्तस्वप्नावबोधस्ययोगोभवतिदु:खहा||**Bhagvat Gita6.17||

Just enough diet, work, sleep, wakeful state, entertainment or sport will remove the grievances. This principle of a moderation in every aspect of life is to be followed.

**आयुःसत्त्वबलारोग्यसुखप्रीतिविवर्धनाः।रस्याः स्निग्धाः स्थिरा हृद्या आहाराः सात्त्विकप्रियाः**।।Bhagvat Gita 17.8।।

Sweet fresh fruits and vegetables (raw or properly prepared), whole grain and legumes, fresh dairy products, nuts, natural sweets such as honey and dates (minimizing refined sugars), and nominal amount of fat from dairy or vegetable sources only was advised to be added in his diet. It was instructed that prepared foods should be combined and cooked in a manner that retains or enhances their nutrients. Food should be aesthetically pleasing to the eye and tasteful to the palate (mildly seasoned), and agreeable to the body's constitution. The food is simple, bland and boiled one. Pickles, papad or spices were strictly prohibited.

#### 2.7.3 Regimen

Few points were specifically suggested to add in his daily routine:

1. To be awake by 5 am in the morning.
2. To avoid late night works, movies, etc and go to bed up to 10 p.m.
3. To minimize the use of electronic gadgets like mobile, t. v., laptop, tab etc and to reduce screen time.
4. To study spiritual or motivational literature of his choice.
5. To maintain a personal diary to write down related to his feelings and daily routine.

#### 2.7.4 Progression of the practice

**Session 1 -** Postural correction/correct method of practices.

**Session 2**- Connecting with the feeling and sensation.

**Session 3**- Developing Breath awareness and synchronization of breath with movement.

**Session 4**- *Pratyahara* and visualization, developing overall awareness.

**Session 5**- Practice of the suggested module, stillness of thoughts, feeling calmness.

### 2.8 Outcome measures

#### 2.8.1 Liebowitz Social Anxiety Scale (LSAS)

It is a tool to evaluate the cases of Social anxiety.

**Fig. 2:**
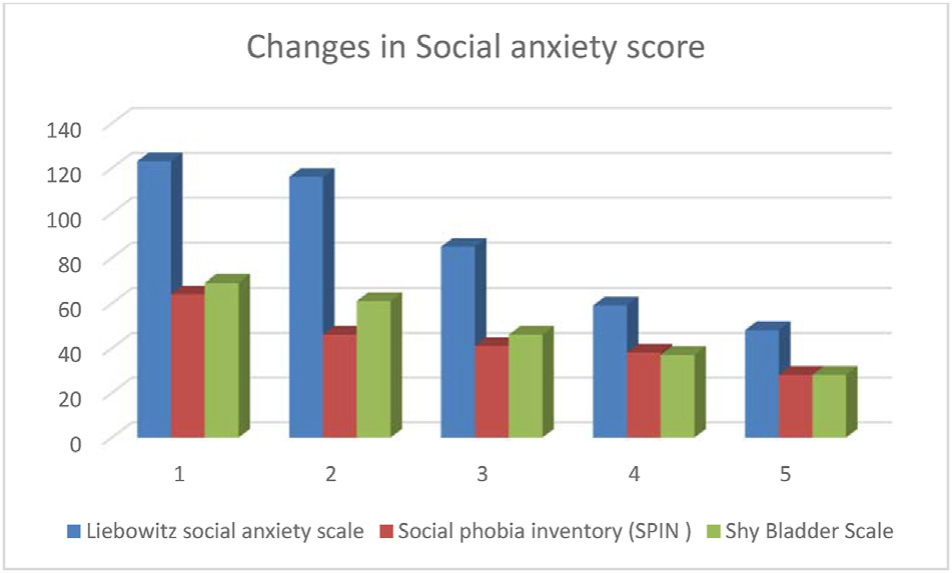
Changes in Social anxiety score.

Progressive improvement in symptoms was found in varied symptoms which restricted the day to day living of the patient. There was significant stress reduction and feeling of relief reported by the patient. Initially the patient had score of 123 which was reduced up to 75 (60.97%) at the end of this study (See:Fig. 2: Changes in Social anxiety score, and Table 2 Changes in Social anxiety score and Quality of life).

**Table 2:** Changes in Social anxiety score and Quality of life.

S.N	Name of score	B.T.	A.T. (f1)	(f2)	(f3)	(f4)	D	% change
1	Liebowitz social anxiety scale	123	116	85	59	48	75	60.97% reduction
2	Social phobia inventory (SPIN)	64	46	41	38	28	36	56.25% reduction
3	Shy Bladder Scale	69	61	46	37	28	41	59.42% reduction
4	WHO-QOL-BREF							
	Physical Domain (D1)	44	51	55	63	69	25	56.81% improvement
	Psychological Domain(D2)	50	52	59	63	67	17	34% improvement
	Social relationship Domain (D3)	52	53	56	57	58	6	10.34% improvement
	Environment Domain (D4)	43	46	50	52	55	12	27.91% improvement
5	Social anxiety	very severe	very severe	severe	Moderate	Mild		very severe to mild

#### 2.8.2 Social Phobia Inventory (SPIN)-

At the beginning of the intervention the tool measured very severe SAnD (Score = 64); after 6 months of Yoga module performance the SAnD came down to a mild score (Score36) after the marked reduction of 56.25%.

#### 2.8.3 Shy bladder scale (SBS) score

Initially it was found 69, which remarks as severe condition of shy bladder. After six months of regular yoga and meditation practice, this situation was remarkably improved up to 59.42%.

#### 2.8.4 WHO quality of life questionnaire- WHO QoL- BREF

**Fig. 3:**
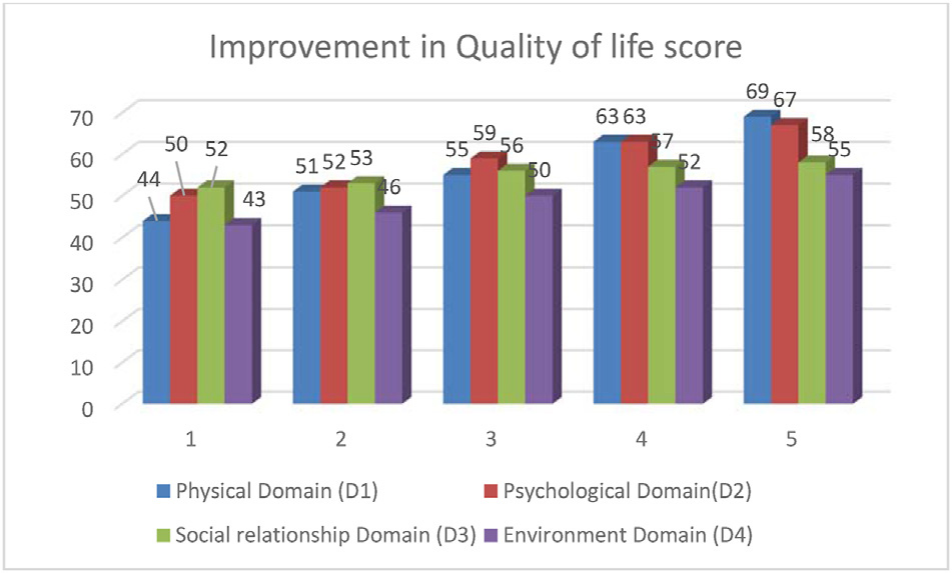
Improvement in Quality of life score.

In our study, we found that yoga and meditation practices in this case of Social anxiety improved WHO QoL- BREF score in each domain. We found 56.81% improvement in physical domain (D1), 34% in psychological domain (D2), 10.34% in social relationship domain and 27.91% in environment domain (D4) (See:Fig. 3: Improvement in Quality of life score).

### 2.9 Follow up

Regular follow up visit of patient for Guided Yoga Module and data collection was scheduled after 15th day, 2nd month, 4th month and 6th month. On every visit, his complaints were recorded as per the standard protocol followed in yoga OPD. The complete session of 30 min was repeated every time to motivate and to properly train the patient in yoga practice. After each practical yoga session, assessment was done using the pre-defined tools on every event. Complaints of patient were also recorded in his own words to ensure the changes in his symptoms.

From recent follow-up, as per patient and his wife, there is no aggravation of symptoms of Social anxiety and Shy bladder syndrome; and improvement was seen in all his symptoms. He started taking part in day to day activities without any hesitation, which was not the case before adopting Yoga therapy.

## 3 Patient's perspective on treatment received

“My problems like phobia of darkness, shy bladder syndrome, and social phobia with other anxiety issues were started since childhood when I was studying in class eight. I could not sleep alone in a room even if my parents, brother, sister are at home; and if alone in house then I could not sleep due to phobia even with lights switched on. The second major problem which I faced was that, I could not urinate in open area or in public toilet; which was mentally painful to me. Several other anxiety issues such as social phobia, was also creating problem in life. I started my treatment in psychiatric department of hospital since august, 2017. Doctors were prescribing me antidepressants and sleeping pills. I was taking medicines regularly, but could not get any improvement. I had planned to leave the treatment in between as I was not getting any relief. Meanwhile I joined my job, but in job training also I faced much problem. So, I continued my treatment. I was suggested for Yoga therapy and psychological sessions along with medication. As Yoga therapy progressed (half an hour tailored session twice daily) I stated getting relief during the first 15–20 days of Yoga treatment. Now I could sleep alone at night with lights on; this was far better than earlier condition. I feel more confident”.

## 4 Discussion

The tailored session for this particular case was prepared by yoga expert, keeping in mind all the symptoms and desired yoga results. According to *Hath yoga pradeepika*, the practice of *dirghshwas pranayama* (deep breathing)*, nadi shuddhi* (alternate nostril breathing) and *bhramari* (humming bee breath) relieves mental stress and anxiety and helps to ease anger. Concentration and mental focus also improves. *Simhasana* (lion's pose) is very helpful to externalize introverted people. *Veerasana* (hero's pose) stabilizes the energy flow to the uro-genital organs and enables control of the energy in pelvic region. It also increases self-control and strengthens the body. *Bhadrakonasana* (butterfly pose) is helpful to induces *moola bandha* and *vajara nadi* and tones the pelvic part, alleviating disorders related to those organs. *Markatasana* (spinal twist pose) is good to relax both body and mind. *Vipareet karni* (reversing attitude) is meant for healthy body and can channelize the body fluid towards brain. *Trataka* (concentrated gazing) benefits in depression, insomnia, allergy, anxiety, postural problems, poor concentration and memory [12]. This tailored session was found effective in boosting the confidence level of patient and enhancing the urinary flow in anxious situation. This protocol was well tolerated and effective in this case.

In this case report, pre and post data was obtained based on a daily yoga routine advised for the duration of 30 min. Follow up visit was scheduled every 15 days for assessing the progress and performance of the Yoga practices taught. A diary was maintained by the patient to track his own daily routine and to write down any queries, progression and comments. Changes in the symptoms explained were assessed on the three different tools used for subjective parameters.

Beside yoga, some other non-pharmacological approaches have also been found effective in social anxiety disorder. In a randomized control trial conducted on 93 patients suffering from General anxiety disorder has shown that 8 week intervention of mindfulness meditation reduced significantly Hamilton Anxiety Scale (HAM-A), Clinical Global Impression of Severity and Improvement (CGI-S and CGI-I) and Beck Anxiety Inventory (BAI) (all p < 0.05) [8]. Trevizol AP et al., performed a single case study of social anxiety disorder treated through neuro-modulation by Trigeminal nerve stimulation (TNS) and found improvement after ten sittings [9] They also used Social Phobia Inventory (SPIN) and Liebowitz Social Anxiety Disorder Scale (LSAS) along with Montreal Cognitive Assessment (MOCA). In another case report, the adult-focused Clark and Wells (1995) model of social anxiety disorder (SAnD) was also used to guide clinic-based treatment for single case study in adolescent in social anxiety co-morbid with panic and agoraphobia and also found improvement in Revised Child Anxiety and Depression Scale (RCADS) and Session Rating Scale (SRS) [10]. Non-pharmacological approaches to treat Social anxiety disorder should always be preferable and tailored or individualized yoga and meditation therapy may bring the better outcome.

## 5 Conclusion

Progressive improvement in the score measuring SAnD was noted in this patient. There was reduction in the severity of the issues faced by the patient such as bladder shyness and agoraphobia.

## Author's contribution

SM and DJ both the authors were involved in concept design, literature search, data compilation, interpretation, and preparation of the manuscript. SM conducted all the yoga sessions. Both the authors reviewed and approved the final article.

## Declaration of conflicts of interest

The authors declare that they have no conflict of interest.

## Funding

NIL.
